# Ground State Configurations and Metastable Phases of Charged Linear Rods

**DOI:** 10.1021/acsomega.2c08060

**Published:** 2023-02-06

**Authors:** Tor Sewring, Martin Trulsson

**Affiliations:** † Theoretical Chemistry, Lund University, 221 00Lund, Sweden

## Abstract

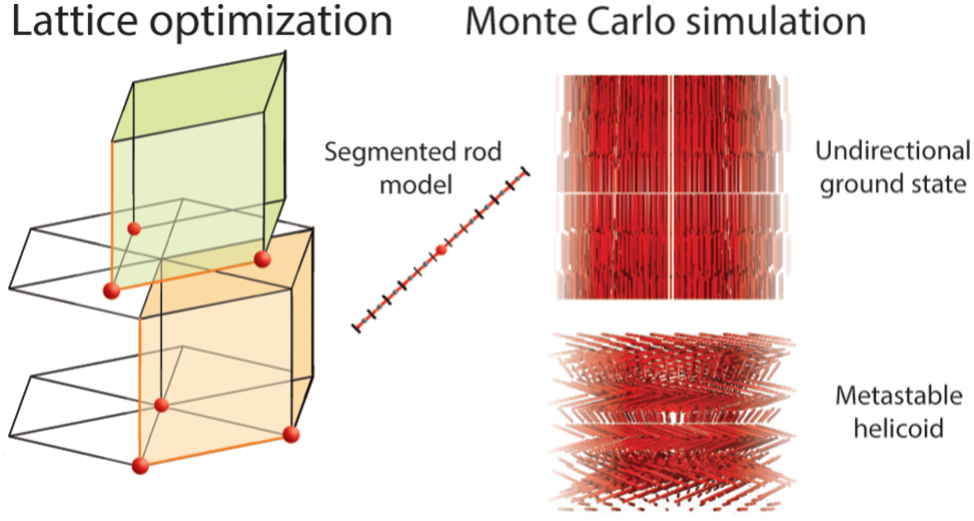

This computational study investigates the energy minimum, that is, ground state, of suspensions of monodisperse (single-component) charged linear rods at various densities and screening lengths. We find that closed-packed unidirectional configurations have the lowest energies for all studied cases. We further specify the lattice parameters for these crystalline structures. In addition, we identify a few metastable phases, including heliconical structures. These metastable heliconical phases are composed of hexagonal smectic C layers with particle orientations forming a conical helicoid with a short pitch of three layers. We evidence this by zero-temperature Monte Carlo simulations starting from an energy-minimized hexagonal cholesteric configuration, which rapidly transforms to a heliconical phase. Furthermore, this heliconical phase is remarkably stable even at finite temperatures and melts to a disordered phase at high temperatures. Finally, we conduct simulations at room temperature and conditions typical for cellulose nanocrystal suspensions to study the onset of nematic order and compare our results to available experimental data. Our findings suggest that electrostatics play an important role in the isotropic/anisotropic transition for dense suspensions of charged rods.

## Introduction

Anisotropic phases of molecules and colloidal particles in liquids have attracted increasing research efforts dedicated to the understanding of the underlying physics ever since the seminal work of Onsager on the isotropic–nematic phase transition for anisometric particles.^1^ Not only do Monte Carlo simulations^2^ verify Onsager’s arguments that hard rods form nematic phases when compacted, that is on increasing the density of the rods, they also detect transitions to smectic phases^3^ followed by hexagonal crystalline structures. Such phases have also been found in experiments, including a hexagonal columnar.^4^ This illustrates that excluded volume alone suffices for the formation of a wide range of structures, from the nematic, in which the particle orientations are aligned along a phase director while their spacial positions lacking order, to the further positional ordered smectic where distinguishable layers of rods form, all the way to hexagonal positional ordering.

For achiral molecules having bent-core shapes, heliconical phase structures such as the twist-bend nematic and heliconical smectic C have been observed experimentally,^5−7^ where the molecular length and curvature dictates which phase preferentially emerges. While the twist-bend nematic phase was found for the short molecules, the longer ones formed smectic layers separated by about one molecular length.^7^ The observed pitch length of the helical twist was short, only a few molecular distances, and the conical tilt angle was found to be about 10°. More recently, the twist-bend nematic phase was also reported in *NPT*-Monte Carlo simulations of hard banana-shaped (curved) particles,^8^ predicted already two decades ago^9^ and found in simulations of particles interacting via soft potentials.^10^ While this phase was found to be thermodynamically stable for curved particles, for kinked particles, such as the bent-core types, it was only so at sufficiently high aspect-ratio.^8^

Beyond the primary shape of the particles, further attributes may result in an even richer phase behavior depending on the conditions in the system. Charges on rod-like particles in aqueous media introduce long-ranged electrostatic interactions, which can render the phase behavior dependent on electrolytic properties such as salt concentration and pH. Not only may the increased interaction range influence the orientational order,^11^ but the positional as well, resulting in the formation of columnar hexagonal phases^12^ even at dilute conditions.^13^ Cellulose nanocrystals, which are highly charged and with high-aspect-ratio in their dimensions, typically exhibit a cholesteric phase structure in colloidal dispersions in aqueous solutions, although biphasic (isotropic/anisotropic) at the concentration levels in the order of a few wt % or vol %, for example, volume fractions of 2.5–6.5%.^14^ However, by subjecting a dense partly gelled suspension (∼10 wt %) to shear forces, it is possible to align the particles in an (achiral) nematic order. When the system is allowed to relax from the shear stresses, intermediate structures have been observed resembling the twist-bend nematic phase prior to reaching the cholesteric phase.^15^ It was proposed that, instead of a twisting/untwisting process between the nematic/cholesteric phase, the transition occurs through an intermediate (nonequilibrium) conical helicoid. This is interesting because by modulating the external forces acting on the particles, here via shear, three orientational patterns were observed for these colloidal particles, which to a first approximation are charged rods (at the increased resolution, characterisations of particles indicate more complicated structures^16,17^). Helical charge distributions on rod-like particles have been addressed as the origin of the cholesteric phase in studies using Monte Carlo simulations.^18^ Rods with a uniaxial charge distribution have also been considered,^19^ but it was found, via a second-virial Onsager free energy functional, that such a charge distribution was insufficient to stabilize a cholesteric phase, that is, induce chiral symmetry breaking, at least on the level of two-body correlations. However, the authors also pointed out that higher numbers of body-correlations (say three) remains to be studied as well as nonlinear effects of electrostatics.

Long-range electrostatics can render a large number of particle interactions even beyond the first neighboring shell, which influences a particle’s state. Thus, the most favorable configurations of a system may be far from trivial. In this study, we investigate to which extent many-body effects may favor some of the different types of phase structures previously mentioned, merely by considering long-ranged electrostatic interactions of monodisperse linear rods, without excluded volume. We focus on this simple case as to investigate if electrostatic many-body effects alone is sufficient to give rise to chiral phases. As opposed to the effect of excluded volume, where entropy controls the phase behavior and which has been extensively investigated already, the literature is scarce of papers addressing the opposite questions: ”What structures do the system seek if we let it be dominated by energy alone?” and ”Could electrostatic alone be sufficient to render chiral structures?” Unlike many simulation^18,20^ and theoretical^11,12,19^ studies, we focus on the ground-state of this problem, being the state favored at low temperatures (ultimately zero) and strong electrostatic interactions, rather than on finite temperatures where the thermal energy is comparable to the electrostatic interaction-energy. Guided by the results for these underlying energy structures, we then discuss the phase diagram of cellulose nanocrystal dispersions at experimental conditions, particularly regarding the isotropic/anisotropic phase transition.

## Model and Computational Methods

### Segmented-Rod Model

We model the electrostatic interaction between two rigid rods *p* and *q*, having equal numbers of effective elementary charges, *Z*, through a segmented-rod model. Each rod is composed of *M* equal segments, each with a charge of *Ze*/*M*, where *e* is the elementary charge. The reduced interaction energy *βu*_*ij*_ between two segments *i* and *j* is given by the anisotropic Yukawa potential for rods:^21^
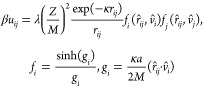
1where ***r̂***_*ij*_ is the distance unit vector, ***v̂***_*i*_ and ***v̂***_*j*_ the two orientational unit vectors, *a* the rod length, *r*_*ij*_ the center-to-center distance between segments, and κ^–1^ and λ are the Debye screening and some arbitrary length scale (the Bjerrum length *l*_B_ for energies in units of *k*_B_*T* or the Debye screening length for reduced energies), respectively. This potential (eq 1) is derived for far-field electrostatic interactions, where the separation between the rod-like units (here segment separations, *r*_*ij*_) are assumed to be much larger than κ^–1^ and the segment length *a*/*M*, where a large *M*, thus, increasingly favors the latter condition. A similar approach has previously been used to study the phase diagram of monodisperse charged disc-like particles.^22,23^ The total interaction energy between two rods *p* and *q* is given by
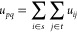
2

We ignore the self-energies of the rods (that is, *u*_*ss*_ and *u*_*tt*_) and consider, furthermore, that the rods are volumeless, equivalent with infinite aspect ratio (length-to-width). Since the interaction between rods are purely repulsive via a Yukawa potential, the rods never overlap due to the high energy penalty for doing so. For selected cases, we further decompose the interaction energy in intra- and interplane energies, where the intraplane energies are defined as the energy between a central rod and all other rods in the same layer and the interplane as the energy between one central rod and all other rods belonging to other layers.

### Crystal Lattice Optimization

Our approach in studying relevant phase structures was to postulate possible crystal lattices and, for each lattice, find favorable lattice dimensions and orientations of the particles by energy minimization (corresponding to *T* = 0, that is, with minimum entropic effects). The lattices were constructed as hexagonal layers in the *xy*-plane stacked *N*_stack_ times along the *z*-axis, with the particle separation distance *l*_*xy*_ in the hexagonal layer and the layer separation *l*_*z*_ between the planes. In addition to the stacked hexagonal layers on top of each other, two variants of the stacks were also constructed where the hexagonal layers were shifted periodically in accordance with hexagonal close-packing (hcp) or the face-centered cubic (fcc), (sequence A-B-A-··· and A-B-C-A-B-···, respectively). The latter lattice is similar to a modified fcc-lattice with a prismatic unit cell used elsewhere,^24^ with variable *l*_*xy*_ and *l*_*z*_ parameters. The lattices will be referred to as AAA, ABA, and ABC, respectively. There are also corresponding cubic lattices, but for hard rods at high packing fractions, the favored positional ordering corresponds to the hexagonal lattices described above.^2^ We verified that, indeed, optimized cubic lattices (AAA and ABA, akin to simple cubic and body-centered cubic (bcc), but square-based and with variable height) render higher energies than the corresponding optimized hexagonal structures, hence, we have omitted the cubic variants in our study.

For each data point the number density ρ = *N*/*V* is fixed (as well as κ and *a*), and the optimum (lowest energy) is searched for by varying *l*_*xy*_ (and thus also *l*_*z*_) and rod orientations, which will be describe below. The number density is given by the reduced particle density ρ* that is being considered, with *a* as the length scale:
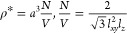
3Similarly, we report our lengths in units of *a*, that is, inverse screening length in units of κ* = *κ a*, and the lattice parameters as *l*_xy_^*^ = *l*_*xy*_/*a*, and *l*_z_^*^ = *l*_*z*_/*a*.

**Figure 1:**
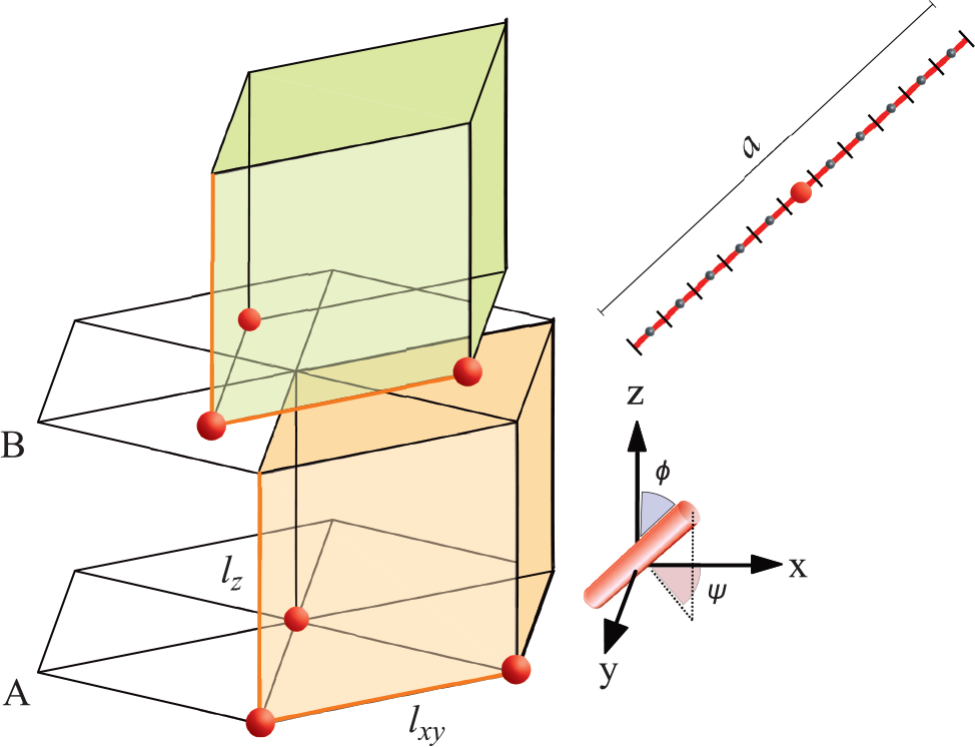
Schematic representation of hexagonal layers (A and B), lattice dimensions of the unit cell (*l*_*xy*_ and *l*_*z*_), angle definitions (tilt angle ϕ and azimuthal angle ψ), and the segmented rod of length (*a*).

The orientational unit vector of each particle in spherical coordinates can be expressed as
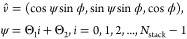
4where *N*_stack_, as previously mentioned, is the number of layers in the crystal stacked along the *z*-axis, ϕ is the polar, and ψ is the azimuthal angle, respectively (see also Figure 1). To describe helical phases, the latter can be parametrized in terms of a twist angle Θ_1_ and a start angle Θ_2_. The latter is used in order to not restrict values of ψ to zero for the first layer or, in the case Θ_1_ = 0, for all layers.

The energy per particle for the crystal lattice system can be estimated by computing the interaction energy of a central particle in a (hexagonal) plane with all its close neighbors within a truncation, and repeat this for *N*_stack_ planes. The segment-to-segment pair potentials were truncated at a reduced distance *n*_trunc_/κ* + 1.5 (ρ*)^−1/3^, where *n*_trunc_ is a positive integer. Thus, the particle–particle interactions could be truncated at reduced center-to-center separations exceeding 1 + *n*_trunc_/κ* + 1.5 (ρ*)^−1/3^. The two latter terms control so that sufficient interactions are included, both when the screening length is relatively long or short compared to the particle density, respectively. This truncation also defines the minimal system size, such as the number of neighbor shells a central particle have in the hexagonal layer, see Figure 1. In the lattice optimizations, *n*_trunc_ was set to 10 (see the sensitivity of *n*_trunc_ on the energy in Figure S1 in the Supporting Information (SI)). Given the above truncations, we typically obtain system sizes of ∼1000 rods for κ* = 50 and ∼10 000 for κ* = 5 at ρ* = 10, as examples of short and long interaction lengths, respectively. For all the reported energies we have discretized the rods with *M* = 15. While there is a dependence on *M*, the relative energies between the states stays almost fixed (see Figure S1 in SI).

We focus our investigations in the parameter span ρ* ≥ 1 and κ* ≥ 5. Typically below ρ* < 1 (and κ* > 5) the rods do not interact strongly enough to mutually orient themselves and taking an energy average over all orientations gives the same energy as having specific orientations. Lower κ*-values renders our computations time-consuming, and for 100 nanometric rods this corresponds to κ^–1^ > 20 nm, corresponding to a <0.3 mM monovalent salt solution in aquouse conditions, that is, an almost salt free condition. Compared with typical nanocrystal cellolose suspensions, we furthermore see that a typical isotropic-to-nematic transition occurs well above ρ* > 1 (see section Discussion in the Context of Cellulose Nanocrystals).

### Monte Carlo Simulation

Selected configurations from the crystal lattice optimization were used as start configurations in Monte Carlo (MC) stability tests (*NVT*-isochoric-ensemble) and for scanning of the nematic onset. The simulation box was cuboid and periodic boundary conditions were applied in all three dimensions. The purpose of the stability tests was to investigate the (meta-)stability of the various crystalline configurations, either at *T* = 0 or a finite temperatures, but also to allow for the possibility of finding new phase structures other than the a priori chosen crystals or transitions between structures. The *T* = 0 simulations are, in principle, stochastic (local) energy minimization trials where only moves that lower the energy are accepted, while, unlike the former, finite-*T* simulations obey detailed balance and mimic microscopic thermodynamics. If the configuration remained stable during a test, or if the orientations and positions of rods only became thermally broadened about the starting configuration at finite *T* and recrystallize after quenching, then the system is regarded as (meta-)stable and otherwise unstable. Stability checks were performed by fixed-temperature runs with single-particle displacement and rotations, as well as combined displacement-rotations, which were used with constant parameters throughout each stage. The displacement parameters for each stage were adjusted such that the acceptance rate was 20–50% for the above-mentioned moves, achieved during 300 MC steps (parameter-adjustment for every 10^th^ MC step) prior to the stage with fixed parameters. The adjustment and following constant-parameter stages constituted a block of MC steps which was repeated sequentially in the simulation to maintain preferable acceptance rates throughout the process. Additionally, displacement of the box dimensions at conserved volume was used (that is, isochoric displacements), where a random box length was selected and displaced randomly within ±0.5% of the current length, a second length of the two remaining was chosen at random to be adjusted according to the volume, while the third was conserved. Each MC step includes *N* trial particle displacements and/or rotations (where *N* is the total number of rods) or 9 box dimension displacement attempts.

It should be mentioned that the particles are in principle allowed to come infinitely close (in terms of Yukawa sites from different particles), because no finite volume is attributed to a particle. Nevertheless, we make the same argument as for a similar model used in Brownian dynamics simulations,^12^ that short site–site separations (*r*_*ij*_) are likely to result in unfavorable configurations. Thus, the model becomes increasingly accurate as the screening length increases compared to, for example*,* the radius of a cylindrical particle. For investigation of the isotropic-to-nematic transition, our MC simulations were always initiated from the ground state configuration (in our cases a closed-packed unidirectional configuration) which we then equilibrated for at least 1.47 × 10^5^ MC steps. *M* was set to 15 for all zero-temperature MC simulations, while it were 11 and 7 for the finite-temperature and isotropic-to-nematic transition simulations, respectively. *n*_trunc_ was 10 in all cases except for the nematic-transition simulations where it was 8. *M* was reduced for the finite-*T* simulations to reduce the computational cost, hence, allowed for longer simulations, especially for the isotropic-to-nematic transition simulations (where also *n*_trunc_ was adjusted). The compromise on the accuracy of the energy is deemed acceptable for the purposes of those simulations, judging by the minor energy differences in Figure S1 in SI.

Besides the energy per particle we also study the nematic and biaxial order parameters. Those order parameters are defined as the largest eigenvalue and the difference between the two subsequent, respectively, of , where ⊗ is the dyadic product and *I* is the corresponding identity matrix.^25^

## Results and Discussion

### Energy-Minimized Crystals

We postulate that the optimum zero-temperature configurations will adopt a well-defined crystal structure due to the long-range electrostatic interactions. Given that, we need to find the ground state, characterized by the lowest energy, for a given ρ* and κ*. Hence, for each (ρ*, κ*)-pair we need to optimize *l*_xy_^*^, ϕ, Θ_1_, and Θ_2_.

#### Lattice Parameter Energy-Landscapes and Near-Neighbor Contributions

**Figure 2:**
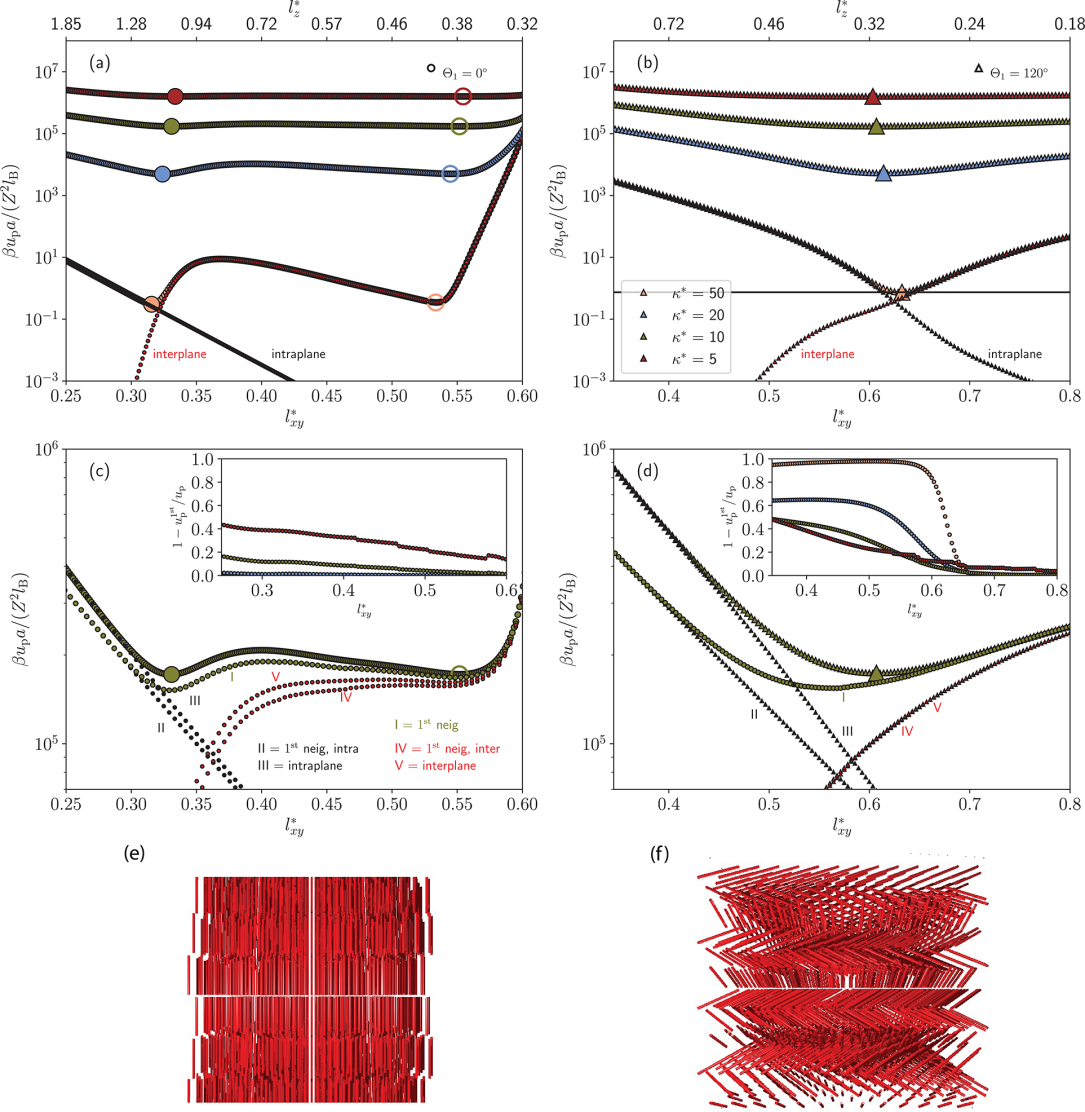
Energy per particle as a function of the reduced lattice parameter *l*_xy_^*^ keeping Θ_1_ (and Θ_2_) and ϕ constant at ρ* = 10, and at various reduced inverse screening lengths κ*. (a) and (c): the ABC-lattice at Θ_1_ = 0° and ϕ = 0°. (b) and (d): the AAA-lattice at Θ_1_ = 120°, Θ_2_ = 0°, and ϕ = 80°. For each inverse reduced screening curve, full and empty enlarged symbols mark the respective global energy minimum and local minimum (if applicable). For the highest inverse reduced screening length κ* = 50 in (a) and (b), the decomposition of the energy into inter and intraplane (*x*, *y*) interaction contributions are shown. (c) and (d): Decomposition for κ* = 10 with further decomposition into 1^st^-neighbor contributions. Inset: The relative 1^st^-neighbor contributions to the energy (*u*_p_^1st^ is for 1^st^ neigh.), at the various inverse reduced screening lengths. (e) and (f): Typical structures of the energy-minima in (a) and (b), respectively.

Figure 2 shows illustrative results of how the energy landscapes evolve as *l*_xy_^*^ is varied at various κ* at a fixed density ρ* = 10 for two of the studied lattice structures (ABC and AAA) having two different rod orientations structures. ρ* = 10 is shown as an experimentally realistic example and κ* ∈ [5,50] a relevant range, which will be apparent further below in Figure 7. We will henceforth mainly focus on the ρ* = 10 case, but other densities (ρ*= 1, 5, and 20 can be found in the SI). For the ABC-structure with rod orientations according to ϕ = 0°, we typically find two minima, a primary at *l*_xy_^*^ ≈ 1/3 and a secondary at *l*_xy_^*^ ≈ 1/2 (see Figure 2(a)), separated by an energy barrier. For the AAA-structure with tilt angle ϕ = 80° and twist angle Θ_1_ = 120° (and Θ_2_ = 0°), we only find one minimum around *l*_xy_^*^ ≈ 2/3 (see Figure 2(b)). Corresponding *l*_z_^*^-values are ≈1.2 and ≈0.4 for the primary and secondary minima, respectively, of ABC, and ≈0.3 for AAA. Hence, for the primary minimum of an ABC-structure (ϕ = 0°), the rods’ ends of two neighboring layers form new hexagonal structures. The secondary minimum instead shows that rods of neighboring layers pierce through the lattice central hexagonal layer. For the ABC, the piercing from above and below are never overlapping, allowing *l*_z_^*^ to be less than half. This is not the case for a ABA structure, as the two neighboring ends would start to overlap. For the tilted AAA-structure, *l*_z_^*^ is naturally allowed to be less than 1 if the particle orientations are tilted.

Since these two structures are at the same density we can directly compare their energy minima. These are in general close to each other but differ most significantly in relative terms at high κ*. For example, at κ* = 50 the secondary minimum differ by 15% from the ground state, while the AAA structure’s minimum differ by 145% (see also Figure S2 in SI). At decreasing κ* the relative energies decreases (including the energy barrier), but increases in absolute values.

Energies can, furthermore, be decomposed into intra- and interplane energies, where as previously mentioned the intraplane energy comes from interactions between a central rod and rods the plane of a hexagonal layer. Conversely, the interplane part comes from the remaining rods, outside that hexagonal layer. As illustrated by the case at κ* = 50 in Figure 2, we see that the two terms have different behavior as a function of *l*_xy_^*^, where the interplane energy is overall increasing while the intraplane contribution is decreasing for increasing *l*_xy_^*^. This can be expected by the given geometry. However, in the ABC-case the interplane shows a highly nonlinear and nonmonotonic behavior as *l*_xy_^*^ is varied between the two minima. This is a combination of both decreasing distance in *z*-direction, and hence, a larger “interaction” overlap between the layers, as well as an increased spacing in *l*_xy_^*^ which affects the *xy*-spacing in neighboring layers, and thus, increases the distance between the rods’ ends of neighboring layers in the *xy*-plane. For the AAA-case, the interplane energy is monotonically increasing as closest rods’ ends from nearest layers do not change their relative distances in the *xy*-plane.

For large κ* the many-body problem is essentially reduced to one particle interacting with its 12 closest neighbors (6 in intraplane and 6 in interplane, where 3 are above and 3 below) for a close-packed structure (ABC or ABA). For the AAA-case in Figure 2(b) and (d), however, the dominating interactions are less intuitive and will be further discussed below. Generally, we see that the primary minimum occurs when the intra- and interplane contributions are roughly equal to each other. The energy-flanks (at small and large *l*_xy_^*^ values) are well-described by exponentials (linear in the log-lin plots). There are some deviations for structures with Θ_1_ ≠ 0, as the nearest intraplane neighbors are not identical, but rather composed into two types, and do not contribute equally to the energy. The main effect of decreasing κ* is increased energies in absolute terms, even if *l*_xy_^*^ is shifted to higher values for the ABC-structure and in the opposite direction for the AAA-structure.

Decreasing κ* also means that interactions are increasingly long-ranged, rending the second nearest neighbors to contribute to the energy significantly in the shown ABC-example. For example, in the case of intraplane interactions, the 12 secondary neighbors’ contribution starts to be large compared to the primary when κ*l_xy_^*^ becomes small (Figure 2(a) and (c)), that is, at low inverse screening length and/or high densities (as *l*_xy_^*^ ∼ (ρ*)^−1/3^). Figure 2(c) shows that the major contribution for the unidirectional ABC, still comes from the nearest neighbors even at κ* = 5 at ρ* = 10.

At its energy-minimum configuration, the tilted AAA-structure is however much more influenced by the neighbors further away than the first shell for sufficiently high κ* as can be seen in Figure 2(d) and especially in its inset. In fact, the second nearest neighbors dominate almost entirely over the nearest in the hexagonal layer at our highest κ*(= 50). This comes from the fact that the particles almost have their directions parallel to the hexagonal layers at high ϕ (= 80°) and that at large values of κ* the energy is essentially dominated by the segment–segment interaction with the smallest separation. For rods with their directions in the hexagonal layer (and Θ_2_ = 0), the closest separation is between a central rod’s end-segments and end-segments from two of its second neighboring rods (one behind and one in front of the central rod).

A general observation is furthermore that for large κ*-values the interaction is dominated by a few closest segment–segment interactions, while for small κ*-values it is increasingly the rods’ center-to-center distances that matter.

#### Tilt Angle Energy-Landscapes

We now turn to the effect of varying the tilt angle, going from ϕ = 0° (uniaxial) to ϕ = 90°, where the rods are oriented in the hexagonal layers. Due to the symmetry of the rod, ϕ = 180°–ϕ when ϕ > 90°. We have restricted the analysis to the chiral twist angles Θ_1_ = 0°, 60°, 120°, and 180° when the rods were tilted and with Θ_2_ = 0° (or 60, 120°, ...) because these were found to be particularly favorable when scanning Θ_1_ at ϕ = 90° and this is related to the rod positions in the hexagonal lattice, see Figure S3 in SI for further details. Θ_2_ = 0 in all cases further on, if nothing else is stated. Furthermore, simulations (where any angles are in principle allowed) support the stability of some of these specific angles, namely Θ_1_ = 120 for AAA, which will be discussed further in Figure 6 in comparison with data in Figure S7 in SI.

**Figure 3:**
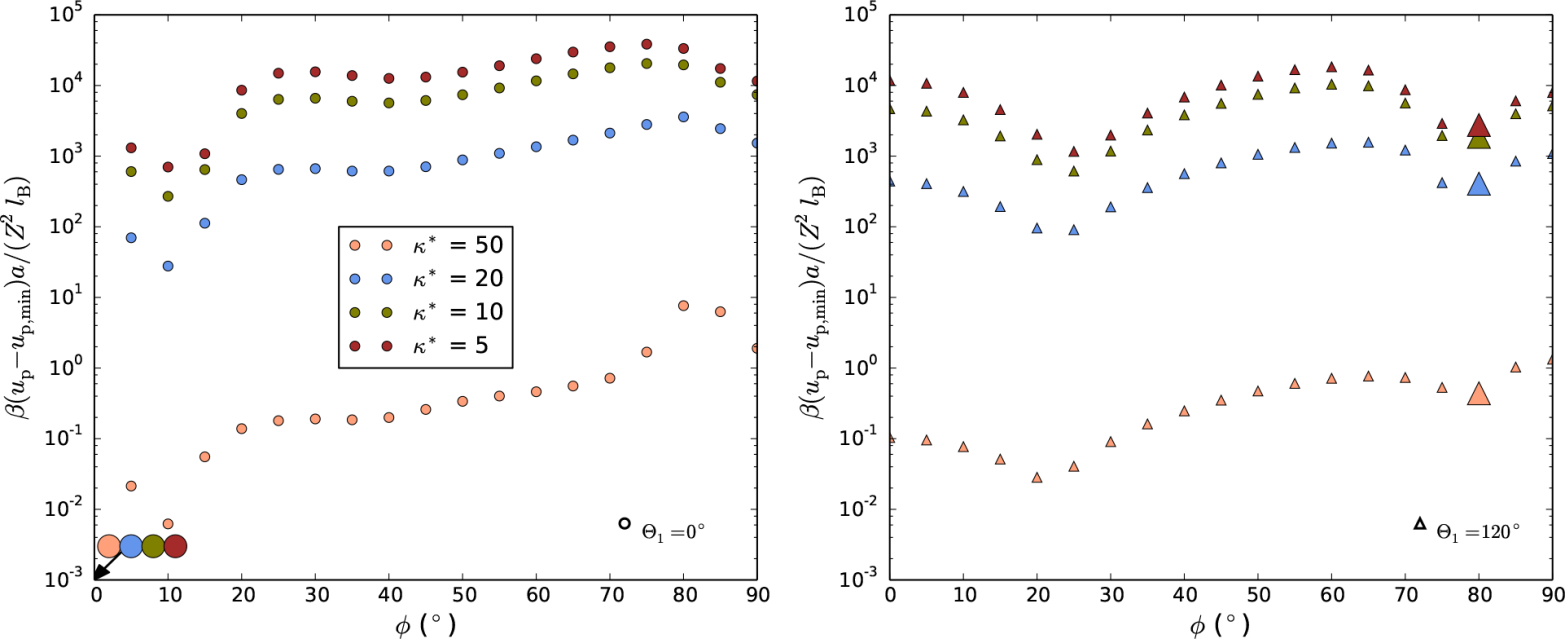
Left: relative average particle energies for the optimal crystal structures (that is, optimized *l*_xy_^*^) at various tilt angles, ϕ, normalized by ground state (that is, ABC or ABA at ϕ = 0) at ρ*= 10. Enlarged symbols correspond to the energy minima in Figure 2.

Figure 3 shows the optimum energies for the previous considered lattice structures (ABC or AAA) and with optimized lattice parameters following the above procedure, for ρ* = 10. We show here the energy difference compared to the optimum unidirectional (ϕ = 0°) ABC-structure. As it turns out, the unidirectional close-packed (ABC or ABA) structures are always lowest in energy for the configurations and densities we have considered, which is also our main result, and we will return to this further below. Figure 3 shows how the energy per particle evolves from this optimum configuration as we tilt the charged rods at this ρ*. From the figure, we see that various local minima appear, where the locations of the highlighted minima correspond to those of Figure 2. The left part shows that for a close-packed structure, the optimal tilt angle is 0°, which, as previously mentioned, holds for both the ABA and ABC lattice. Besides this optimal minimum, we also find local minima at 10° and 90° as well as a shallow one at 45° at low inverse screening lengths.

For a hexagonal lattice (AAA), however, tilted orientations of the rods in relation to the *z*-axis are in many cases more favorable than *z*-oriented for this lattice. In the displayed case, that is, for heliconical structures with a chiral twist angle of 120°, two local minima are found at about 25° and 80° (the latter will be discussed further below in the next paragraph and in the section Metastable Structures). These minima are often less than a few percent higher in energy, depending on κ*, compared to the global one (see Figure S3 in SI).

**Figure 4:**
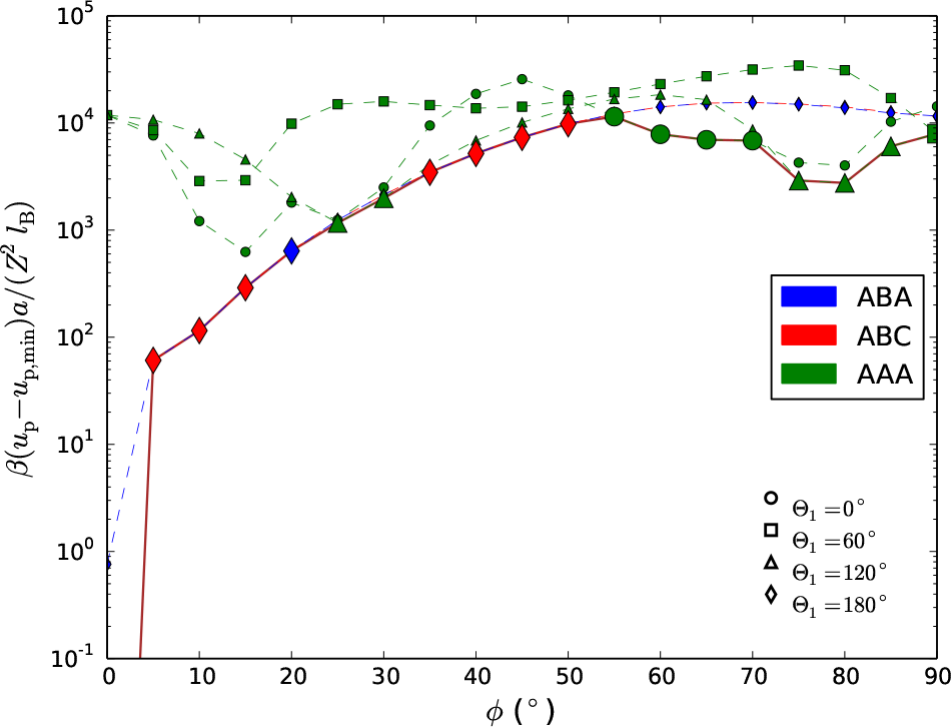
Energies per particle for the optimal crystal structures at various tilt angles, ϕ (ρ*= 10 and κ* = 5). The most favorable configurations are highlighted by enlarged symbols and a full line as guide for the eye, while the ϕ-dependency for selected structures are indicted by smaller symbols and guiding dashed lines. As a comparison we have *βu*_p, min_a/(*Z*^2^*l*_B_) ∼ 2 ×10^6^, that is, the energy differences are typically less than 1%.

To find the optimum structure at a given tilt, we need to compare the energies between all structures. Figure 4 shows the optimized energy as a function of ϕ for κ* = 5 and ρ* = 10 for various considered structures (we only show structures that are the minimum configuration for at least one tilt angle for the sake of visibility).

The overall most favorable structures at these ϕ-angles are highlighted to illustrate an energy landscape along the rod tilting, adopting the most favorable structure. At tilt angles below about 45°, a monotonic decrease in energy was found for decreasing ϕ, toward the ground state structure at ϕ = 0°. In most of the cases in this tilt-range, the structures preferred from the tested crystals are close-packed lattices with Θ_1_ = 180°. Moreover, the slope of the energy profile suggests that those should be unstable, which was confirmed by *T* = 0 MC simulations, where these optimized close-packed structures rapidly relaxed toward the unidirectional ϕ = 0° configurations. It is worth mentioning that simulations of discs, using a model akin to the one used here (eq 1), revealed phase structures with an alternating director (nematic/antinematic) called intergrowth textures.^22,23^ However, in contrast to discs, we find that our one-dimensional rods do not stabilize such structures (that is, with Θ_1_ = 180°).

If instead the range of high tilt angles is considered (55–90°) at ρ* = 10, then the most favorable structures found had an AAA lattice and were of various smectic C-types. In the range ϕ ∈[55°,70°] the rod orientations are unidirectional (Θ_1_ = 0°), but at higher angles, heliconical structures were favored. The results predict two zones which could include metastable structures; one with a (possible) shallow local minimum (Θ_1_ = 0° as mentioned above) and another with a deeper local minimum at tilt angles about 75–80° (Θ_1_ = 120°) (corresponding to the enlarged markers of local minima in Figures 2 and 3).

#### Dependency on the Inverse Screening Length

**Figure 5:**
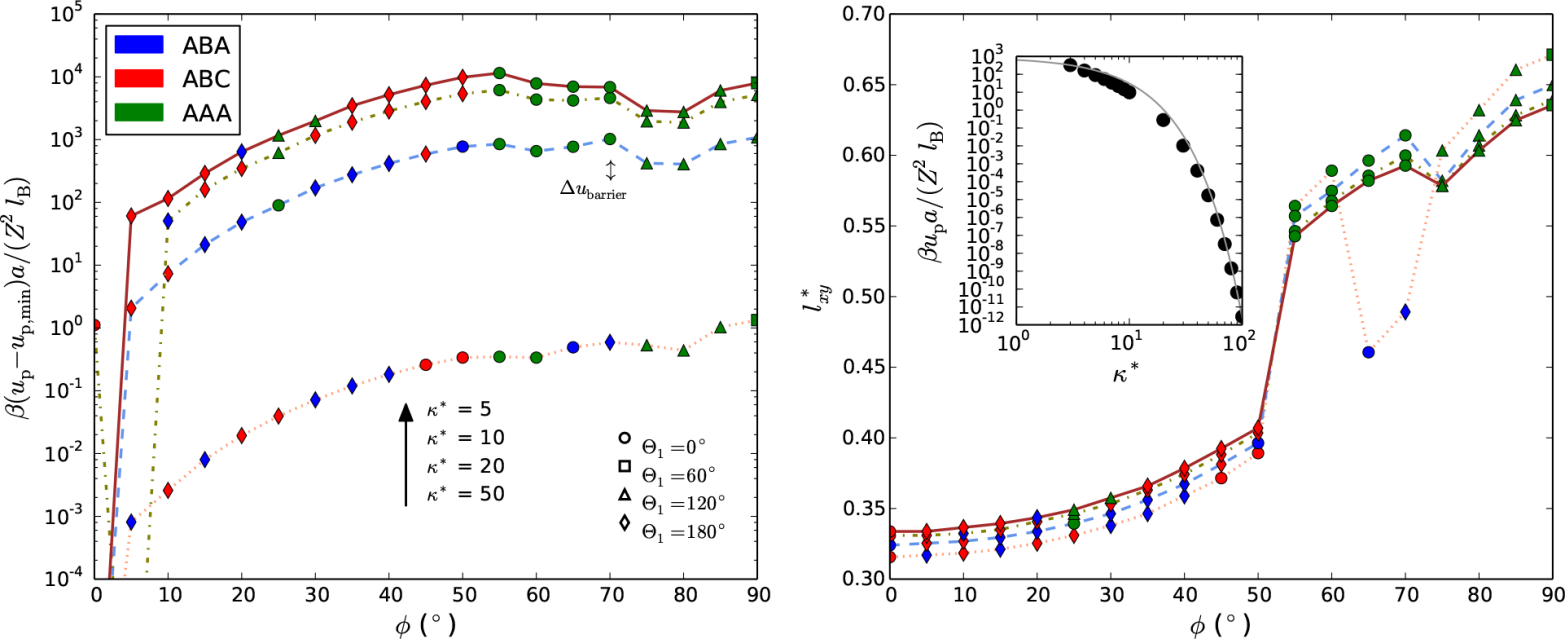
Left: Energies per particle for the optimal crystal structures (ρ*= 10) at various tilt angles, ϕ, and reduced inverse screening lengths, κ*. The full guiding line at κ*= 5 corresponds to Figure 4. Right: The corresponding lattice dimensions *l*_xy_^*^ at various ϕ. Inset: Energy as a function of κ*. Line shows the expression *u*_p_ ∼ *e*^–^κ*l_xy_^*c^, where *l*_xy_^*c^ is characteristic (here 0.34) for this ρ*.

Figure 5 shows similar energy landscapes as in Figure 4, but here at various reduced inverse screening length κ* as a function of the tilt angle ϕ (at ρ* = 10), which all show clear and characteristic local minima. The corresponding figures to Figure 5 for ρ* = 1, 5, and 20 are shown in Figures S4–S6 in SI, which span experimentally relevant values, see Figure 7). Most importantly, we find that the heliconical structures with AAA-lattice and tilt angles close to 75° are locally favorable for all inverse screening lengths. From Figure 5, we can obtain minimum energy barriers that the system needs to overcome to transition from a heliconical to tilted unidirectional configuration and vice versa, and in a similar way from a tilted unidirectional to a unidirectional, *z*-oriented (close-packed). Such values give us an indication of the stability of these phases.

The overall features of the energy landscapes persist over various levels of κ*, although the relative energies to the ground state increases as κ* increases (see left image in Figure 5). In the inset inside the image to the right, the energy increases almost exponentially when κ* decreases at constant density (here ρ* = 10), while *l*_xy_^*^ ≈ 0.34 is almost constant.

In our model, we have neglected the volume of the rods, however, the results indicate that the favored configurations (lowest energy) avoid close distances between segments of rods. The close-packed structures (ABA and ABC) are favored over hexagonal (AAA) for *z*-oriented phases, where the former minimizes short end-to-end distances compared to the latter for smectic structures. The closest segment-to-segment distance is larger than that when one end-segment starts to pierce the hexagonal layer formed from end-segments below, that is ≥ (*l*_xy_^*^√3) /4 ≈ 0.15 at ρ* = 10. We note that for *M* = 15 the reduced segment length is 1/15, less than half of the segment separation, rendering the discretization of rods acceptable (eq 1). When this separation is compared with the reduced screening lengths 1/κ* for κ* = 50, 20, 10, 5, we note that only the last (= 0.2) is larger than 0.15. Yet, increasing *M* would only lower the energy by a few tenth of a percent, see Figure S1 in SI. The penalty of close end-to-end distances can also be observed as the hexagonal smectic C structures (for example, heliconical) are favored over the corresponding *z*-oriented hexagonal (smectic A).

This behavior justifies the neglect of the excluded volume at the investigated density, not the least in our zero-temperature approach. It is also worth noting that close-packed structures (for example, ABC) are favored for anisotropic electrostatics just as for highly compact and ordered hard-rod systems.^2^ The commonality in behavior proposes the effect of the electrostatic interaction to be viewed primarily as a larger effective volume of the rod compared to the actual^11^ (although, there is also a twisting effect^19^). Moreover, simulations (NPT-MC) of charged hard-core spherocylinders of aspect ratio 5 at low temperatures, that is, strongly energetically coupled systems, show that the concentration range of the nematic phase, encapsulated by the isotropic and smectic, is narrowed compared to higher temperatures.^26,27^ The smectic concentration range is, on the contrary, rather extensive, and at high packing fractions and low temperatures, hexatic structures are formed prior to the crystalline phase. Hence, although idealized, our postulated lattice structures (ABA and ABC), having smectic layering and hexagonal positional order, are realistic when electrostatic interactions dominate.

### Metastable Structures

**Figure 6:**
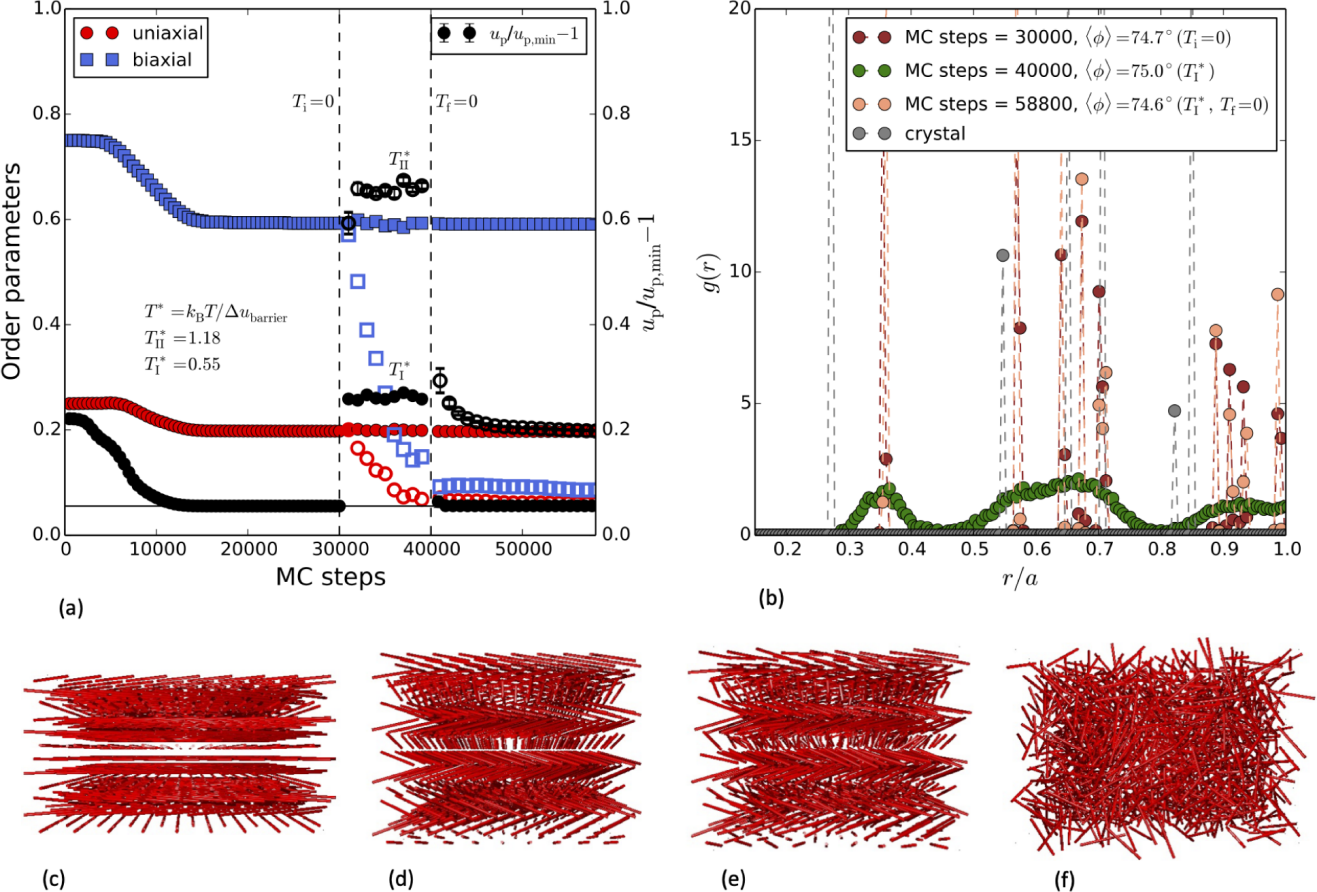
MC stability tests, starting from an AAA-lattice at ϕ = 90° and Θ_1_ = 60°. ρ* = 10 and κ* = 20. (a) Order parameters and relative particle energy difference between the simulation configurations and the ground state reference during the course of the simulation. Various temperatures are employed at various stages. Δ*u*_barrier_ is obtained from the lattice optimization, shown in Figure 5. After 30 000 MC steps, two paths are taken at the temperatures, *T*_I_^*^ →*T*_f_ = 0 (full symbols) and *T*_II_^*^ →*T*_f_ = 0 (empty symbols), respectively. (b) Pair-correlation function at various stages, including the initial crystal configuration. (c) Start config., (d) heliconical config.: *T* = 0, MC-steps = 30 000, (e) heliconical config.: *T*_I_*, MC-steps = 40 000 (after the following *T* = 0 to 60 000 steps, the structure of (d) was regained), (f) glassy state: *T*_II_* followed by *T* = 0, MC-steps = 60 000.

As previously mentioned, we predict possible metastable structures which were further investigated by conducting MC simulations starting from various optimized crystals in the vicinity of the predicted locations of the metastable structures. In Figure 6, we first show that the cholesteric (with respect to orientations) crystalline phase, starting in a AAA-crystal with ϕ = 90° and Θ_1_ = 60° (see structure in Figure 6(c)), is unstable, even at *T* = 0, and relaxes to a heliconical phase with a tilt angle of approximately ϕ = 75° and Θ_1_ = 120° (compare with Figure S7, which depicts the uniaxial and biaxial order parameters as a function of ϕ for a crystalline AAA with Θ_1_ = 120° which coincide with the simulation result, and also with Figure S8, showing an AAA with Θ_1_ = 0°, where, in contrast, the order parameters are unity and zero, respectively). Other optimized cholesteric crystal structures in an ABC-lattice were also found to be unstable, also relaxing to a heliconical phase. One may also notice that among the AAA-structures in the vicinity of ϕ = 75° in Figure 4, those with Θ_1_ = 120° and Θ_1_ = 0° (which will be discussed below) result in local energy minima, while Θ_1_ = 60° render a maximum. This predicts the latter to be unfavorable compared to the former two, in agreement with the simulations.

The heliconical phase is found to be stable at temperatures below the estimated barrier illustrated in Figure 5. After relaxation to the heliconical phase from the cholesteric crystalline, we show this by first heating the system and then cooling it to *T* = 0 (Figure 6). The energy, uniaxial order, biaxial order, and pair-correlation function all collapse on the optimized heliconical configuration after cooling, demonstrating its stability. For temperatures below the melting, the energy increases due to the thermal broadening in positions and orientations. The positional order is, however, broadened considerably more (Figure 6(b)) compared to the orientational as the uniaxial and biaxaial order parameters remain virtually constant even at a finite temperature (Figure 6(a)). The orientational stability of the heliconical structure are also visible when comparing Figure 6(d) and (e). At temperatures above the barrier, the crystal melts to an isotropic configuration and after an instantaneous cooling to *T* = 0, it relaxes to a disordered (glassy) state (see structure in Figure 6(f)) with a higher energy than both the ground state and the heliconical phase and with almost no uniaxial and biaxial order. As an example, if we assume a Bjerrum length of 0.7 nm, a rod length of 100 nm, and a Debye screening length of 5 nm (ρ* = 10) then these barriers would correspond to the rods having a line charge density of 0.6 and 0.4 elementary charges per nm, respectively.

The transition from the cholesteric crystal to the heliconical in Figure 6 also shows that the system may undergo a transformation from one chiral twist angle to another if this is favorable, namely 60° → 120°, but in this case, this necessarily involves a simultaneous adjustment of the tilt angle ϕ and the lattice dimensions (notable from the shift in peak positions in *g*(*r*), in Figure 6(b)). Besides this heliconical phase, we also found the secondary minima of Figure 2(a) and (c) to be stable after *T* = 0 simulations, as well as AAA, Θ_1_ = 0°, ϕ = 10° (see Figure 4), where ϕ only shifts one or two degrees depending on κ*. The same tendency was found for AAA, Θ_1_ = 0°, ϕ = 55°, which can be contrasted to ABC, Θ_1_ = 180°, ϕ = 50°, where ϕ quickly reduces toward zero instead.

Our findings suggest that lattice transformations, for example*,* AAA-to-ABC, in many cases come at very little or no energetic cost, whereas the transformations in the twist angle Θ_1_ can be highly unfavorable unless other transformations can occur simultaneously to aid in the twist-angle transformation (Figure 6). It should also be mentioned that *T* = 0 simulations starting in a AAA-crystal at ϕ = 60° and Θ_1_ = 0° finds the energy well of ϕ ≃ 75°, but remains unidirectional in rod orientations (Θ_1_ = 0°). Notably in the simulations, the system manage to pass the barrier in the range ϕ ∈ [60°,75°]. It does so by displacing the box lengths and tilt ϕ simultaneously in an unexpected manner (see Figure S8 in SI). The lattice finally adopts a distorted hexagonal lattice (AAA), elongated along the *x*-axis (which is also along the rod orientations) on the expense of the other dimensions. It is conceivable that the orientations according to Θ_1_ = 0 (and perhaps also Θ_1_ = 180) enables a favorable box-distortion along the *x*-axis, which is not the case for the heliconical alternative (Θ_1_ = 120) where the box lengths in the *x* and *y* direction prefer to scale equally. Considering these diverse behaviors found, one may expect that numerous arrested states can exist at the density in question (ρ* = 10) and probably at higher densities too. Experimental systems of fd-virus particles with high aspect-ratio (length-to-width = 129) show, for instance, arrested (glassy) states well into the chiral-nematic regime, beyond the isotropic–nematic coexistance, due to long-range electrostatic interactions.^28^ It may also be added that experimental hints of heliconical structures in thermotropic systems of rod-like compounds (tricatenar azobenzenes) have been reported rather recently.^29^ This system is very different from ours, but both indicate that heliconical structures may appear (although rarely and not necessarily stable) even for rod-like particle shapes.

## Discussion in the Context of Cellulose Nanocrystals

Finally, we investigate if our model can predict some typical characteristics of suspensions of rod-like particles. Aqueous dispersions of cellulose nanocrystals transition from the isotropic to an anisotropic phase on densification by means of water removal. Commonly, at the onset of the anisotropic phase, a biphasic region emerge where the extent of the anisotropic phase increases at further removal of water until the system is fully anisotropic.^14,30^ We expect that a primary component governing the transition from an isotropic to an anisotropic phase is the anisotropic electrostatic interaction for charged rod-like particles. It should be mentioned that cellulose nanocrystal systems typically exhibit cholesteric mesostructures rather than nematic. We do not stabilize such structures in our investigation; however, the initial twist angles estimated from experiments are small at transition densities, in the order of a degree.^14^ Hence, on some nanoscale the local director is expected to be not much different from that of a nematic phase, and locally a nematic approximation may be reasonable. Thus, we use the uniaxial order parameter, *S*, from simulations to identify the isotropic/anisotropic transition in the approximate limit of negligible chiral twist angles near the onset of the anisotropic phase.

**Figure 7:**
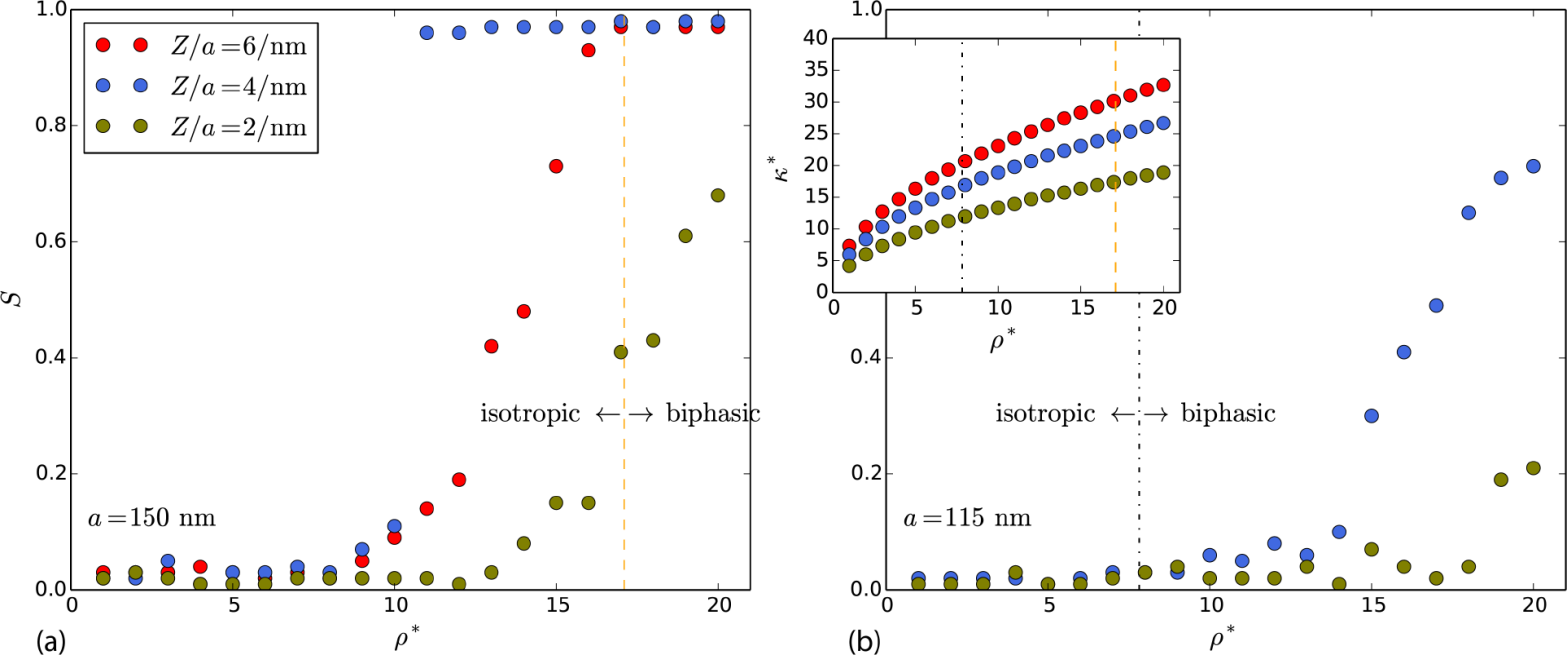
Predicted evaporation curves in the space (ρ*, κ*) and isotropic/anisotropic phase behavior, at various constant valency densities (*Z*/*a*). (a): *a* = 150 nm, corresponding to ref (14). (b): *a* = 115 nm, corresponding to ref (30). The onset of the anisotropic phase from experimental data (see above-mentioned refs) are marked as dashed lines. The uniaxial order parameters, *S*, are obtained from MC simulations.

There may be more particle properties and/or more detailed features that also have significant influence, for example, polydisperisity in size that we so far neglected. Moreover, it has been proposed that cellulose nanocrystal dispersions contain aggregates referred to as bundles which consist of the primary crystallites which are incapable of inducing the macroscopic pitch themselves,^31,32^ and this is supported by our findings. Rather, it is the bundles that propagate the chirality to the meso and macroscopic scale, and their density of them influences the observed pitch. For instance, ultrasonication of a system reduced the population of the bundles in favor of primary crystallites, which increased the pitch. Within the weak chirality limit,^33^ and assuming that the system is dominated by primary crystallites, we may thus investigate to which extent the iso-to-anisiotropic transition can be explained by employing a simple model as used here (representing the crystallites), merely ascribing the particles a length, *a*, and a line valency density, ξ = *Z*/*a* (the corresponding line charge density is given by *ξ e*). Given the experimental conditions in refs (30) and (14) this line charge density is the only unknown parameter in our model and will be estimated below. Furthermore, by assuming that only the dissociated monovalent counterions contribute significantly to the electrostatic screening length (κ^–1^) of the charged rods, an evaporation process can be described by the relation . Along such curves in the (κ*, ρ*) space at constant ξ and *a*, MC simulations were conducted using the ground state structure found in this study as crystalline starting configurations. The crystals were melted to a final temperature of 298 K at a constant Bjerrum length of 0.71 nm, where the temperature was fixed for at least 1.4 × 10^5^ MC-steps. We choose this route rather than starting from an isotropic configuration and cool the system which typically becomes too computationally costly to equilibrate or lead to glassy states. The results are shown in Figure 7 for rod lengths, (a) *a* = 150 and (b) *a* = 115 nm, typical for the experimental systems in refs (14) and (30), respectively, and at relevant levels of ξ. As a reference, the line valency densities based on the experimental sources for fully ionised sites were estimated to 6.0/nm and 4.3/nm, assuming rectangular rods with dimensions (a) 5 × 5 × 150^14^ and (b) 7 × 7 × 115 nm^3^,^30^ with a surface charge density corresponding to the experimentally reported ionizable site/charge densities 0.23 mmol/g cellulose (mass density = 1600 kg/m^3^) and 0.115 *e*/nm^2^, respectively.

Having a finite temperature introduces a new dimensionless unit Λ comparing characteristic electrostatic energy to thermal energy. There exist several ways of defining such a characteristic energy, using either κ^–1^, , or *a* as a characteristic length (or combinations of them). First, we see from Figure 7 that the isotropic-to-nematic transition occurs as the density increases, just as for hard and uncharged anisotropic particles. From Figure 7(a), we do, however, see that at which density this occurs is nonmonotonic as a function of ξ. Having different ξ’s gives both different Λ’s but also different κ*’s. This makes it difficult to predict the experimental (apparent) line charge density. Nevertheless, we can compare parts (a) and (b), as they both follow the same (ρ*,κ*) -curve and only differ in the Λ-parameter. For the three possible length scales, we find that the coupling parameter always scales as Λ ∼ ξ^2^*a* for a fixed (ρ*,κ*) -pair. Hence, the coupling parameters are lower in (b) than in (a) for the same (ξ,ρ*,κ*) -triple, and one expects a transition at higher reduced densities in (b) compared to (a). This is also numerically verified. In ref (14), pH was measured to be 2.3–2.6 in the biphasic region. On our evaporation curve, a pH of 2.6 corresponds to ρ* = 17 and ξ = 2 nm^–1^ and marks the experimental onset of nematic order. The experimental onset is higher than our results for fully ionized sites corresponds to ξ = 6 nm^–1^, and it can not be ruled out that the charges are (partially) saturated (due to nonlinear effects and Manning condensation effects),^34^ rendering an effective lower charge of the rods. Nevertheless, our simulations suggest that an anisotropic phase (nematic in character, at least locally) can be expected in the ρ* range between 5 and 20, in the vicinity of the experimental findings.^14^ For the other case,^30^ however, we find that the simulated order parameter reaches significant nematic order (*S* approaching 0.8 for ξ = 4 nm^–1^) at densities higher than the experimental onset. Given that the system of Dong et al.^30^ is expected to have a lower electrostatic coupling compared to that of Schutz et al.,^14^ it is surprising that the onset occurs at a lower density. This can possibly be explained by an interplay of electrostatic interaction and excluded volume.

It should also be mentioned that experimental systems are widely polydisperse, a property neglected in our simplified and monodisperse model. However, if we consider typical properties, then the experimental systems have aspect ratios (*a*/*d*), where *d* is the width of the rods, of roughly 30 and 17. From Bolhuis and Frenkel,^2^ we estimate the isotropic-to-nematic onset (coexistence) due to purely excluded volume to occur (in our units) at a density of around ρ* ≈ 100 and ρ* ≈ 50 for (*a*/*d*) = 30 and (*a*/*d*) = 17, respectively. Since both densities are considerably higher than the experimental onsets, we thus, conclude that the onset must be largely electrostatic in nature for these systems. This also motivates our approximation, using simple linear charged rods without any volume as a model. To test the idea that isotropic-to-nematic transition for charged rods can be understood by an extended excluded volume due to the electrostatic repulsion, we define new lengths and diameters as *a*′ = *a* + 2 κ^–1^ and *d*′ = *d* + 2 κ^–1^. For the cases in Figure 7, we typically have screening lengths of 10 nm, giving new effective aspect ratios (*a*′/*d*′) around 5. Using the simulated phase diagram of Bolhuis and Frenkel^2^ again, we instead predict a transition at around ρ* ≈ 10 (in our units) and is in fair agreement with both our simulated data and the experiments. However, what causes the reverse trend in the isotropic-to-nematic onset, experimentally compared to theory/simulations, for the two studied cases needs to investigated in the future.

## Conclusions and Outlook

We have studied the ground state of volumeless charged linear rods. We have explicitly searched for the possibilities of having cholesteric, heliconical, or smectic configurations as ground states. Regardless, we always find a unidirectional and closed-packed ground state within our parameter span. However, many of our studied configurations are metastable, including a heliconical phase. Others turn out to be unstable, for example, our postulated cholesteric types, which spontaneously decay to a heliconical phase. It is, however, rather surprising that we find chiral metastable phases, such as the heliconical phase, from purely achiral constitutes.

Our parameter space overlaps with experimental systems of cellulose nanocrystal suspensions, yet we do not find the experimentally reported cholesteric phase to be stable when modeling the particles as linear charged rods. It could however also be important, even essential, to account for excluded volume (including bundles of crystallites), polydispersity, or by internal charge distribution, within the particles or on their surfaces, for its stabilization. This needs to be investigated in the future.

Within the weak chirality limit where the anisotropic phase is approximately nematic locally, we also try to reproduce the experimental isotropic-to-nematic transition (of a evaporation process) with the line charge density as the only fit parameter. This turns out to be a nontrivial task due to that the onset varies in a nontrivial way as a function of the line charge density (as two dimensionless numbers vary simultaneously). Furthermore, while decreasing the rod length, one would expect a transition at a higher reduced density; the two comparable experiments at two different lengths show the opposite trend. Regardless, the results suggest that long-ranged electrostatic interactions ought to be essential in the stabilization of an anisotropic phase, for example, reduces the onset density compared to uncharged hard rods. The question concerning the entropic and energetic nature of the nematic and chiral ordering is highly relevant both from a theoretical and experimental point of view. The latter to understand how to control and alter these structures. For example, it is not unlikely that the chirality of the particle will propagate via the electrostatic interactions as well as the excluded volume. Likewise, one could think about scenarios of the opposite, where electrostatics dampens the tendency to form chiral structures. These questions would naturally be needed to be addressed in the future.

Besides the possibility that internal charge (spatial) distributions or excluded volume stabilize the cholesteric phase or change which configuration turns out to be the ground state, it can be worth to consider whether these highly charged systems necessarily reach equilibrium (or their ground states) at any given condition. The systems can arrive in one of the metastable phases and stay there for the experimental relevant time scales. Which state a system ends up in would then depend on the preparation protocol, for example, by drying or shear flow. For instance, helix unwinding of hydroxypropylcellulose-systems under shear has been shown experimentally.^35^ While others have studied the effect of shear of hard rods and attractive rods,^36,37^ it is theoretically/numerically unknown how electrostatic repulsive rods would behave under shear (including a following shear relaxation) and would be an exciting venture to explore in the future. Other effects that need to be considered in the future, and that we have neglected, are polydispersity in shape, nonlinear effects (that is, solving the full Poisson–Boltzmann equation) and charge regulation effects.

## Supporting Information Available

Energy at various rod segmentation and potential truncation, chiral twist-angle analysis, additional energy-minimized crystals, and additional MC simulation results (*T* = 0) (PDF)
