# Supplementary material for: Ground State Configurations and Metastable Phases of Charged Linear Rods
Source: ACS Omega. 2023 Feb 6;8(6):6040–51. doi: 10.1021/acsomega.2c08060 (PMC9933468; doi:10.1021/acsomega.2c08060)
Supplement: Supplementary file 1 — ao2c08060_si_001.pdf [file ao2c08060_si_001.pdf]

Ground State Configurations and Metastable Phases of Charged Linear Rods

Tor Sewring and Martin Trulsson*

Theoretical Chemistry, Lund University, 221 00 Lund, Sweden

E-mail: *martin.trulsson@teokem.lu.se

Content:

- Energy at various degrees of rod segmentation and potential truncation
- Energy minima (relative energy, Figure 2)
- Chiral twist angles
- Additional energy-minimized crystals
- Monte Carlo simulations, $T = 0$

Energy at various degrees of rod segmentation and potential truncation

For $M \geq 5$, the favouring of phase I over II is consistent, see Figure S. 1. The rank order of the phases is, moreover, independent of the truncation of the range of electrostatic interactions (see dependency on n_{trunc}). The level of segmentation and interaction-range truncation do, however, influence the absolute values of energy, and of course also the computational cost.

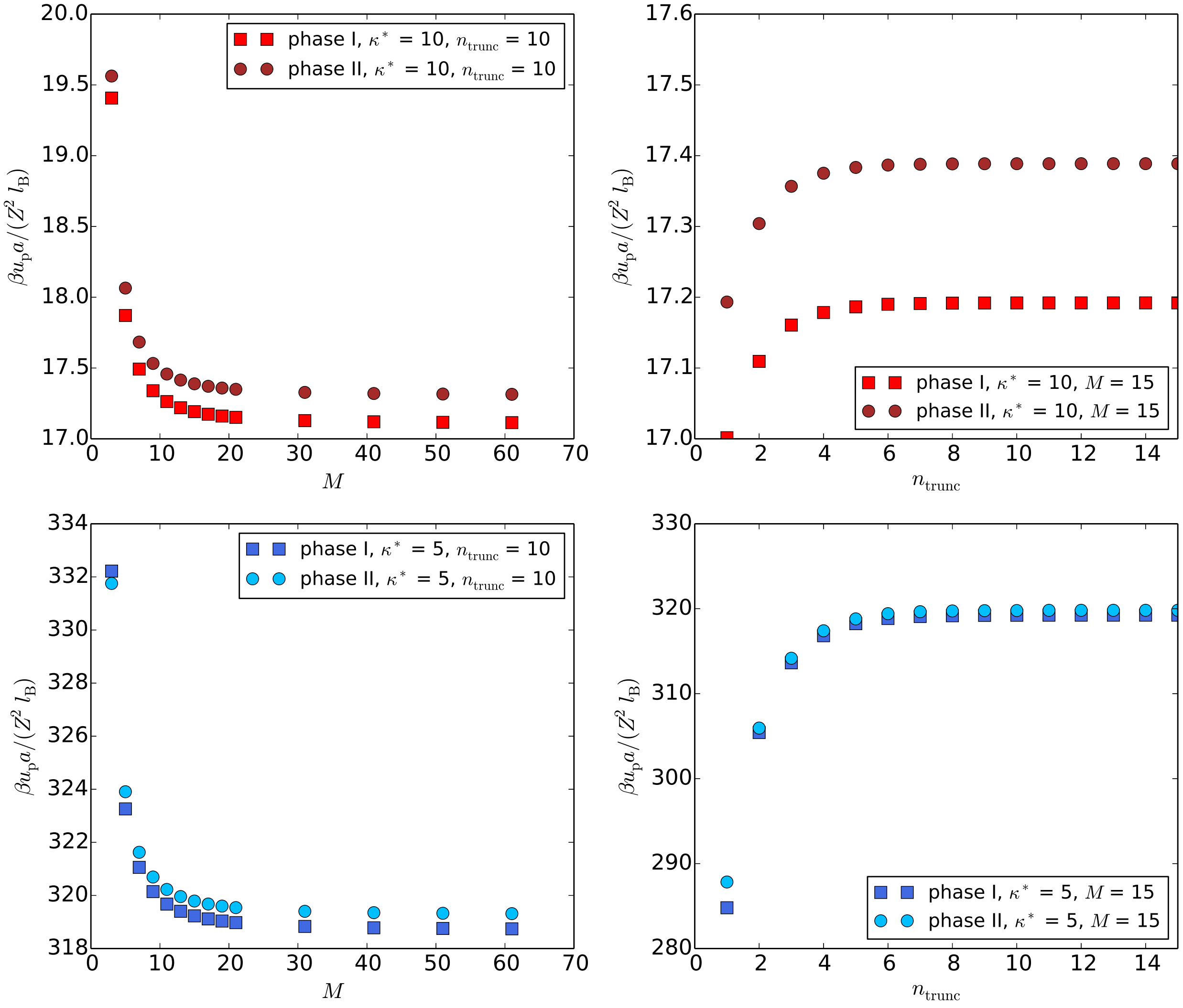

Figure S. 1: Energies per particle at various rod segmentations (M) and potential truncations (n_{trunc}), at ρ^*=10. Phase I=ground state configuration. Phase II=AAA, $\Theta_1 = 120°$, $\phi = 75°$.

Energy minima (relative energy, Figure 2)

Energy profiles in Figure 2, but in relative energy differences.

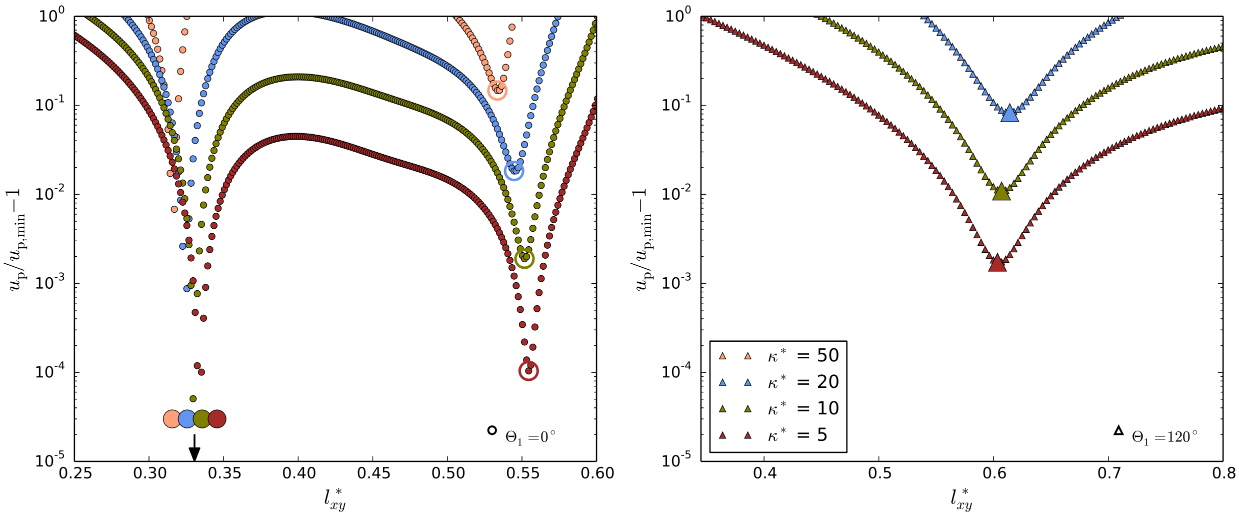

Figure S. 2: The energy difference per particle, relative to the ground state, as a function of the reduced lattice parameter l^*_{xy} keeping Θ_1 (and Θ_2) and ϕ constant at $\rho^* = 10$, and at various inverse reduced screening lengths κ^*. Left: the ABC-lattice and $\Theta_1 = 0°$ and $\phi = 0°$. Right: the AAA-lattice at $\Theta_1 = 120°$ and $\phi = 80°$.

Chiral twist angles

The twist angles $\Theta_1 = 60$ and $120°$, at tilt angle, $\phi = 90°$ generates favorable chiral nematic structures. Their energies are moreover lower than the corresponding unidirectional phase at $\Theta_1 = 0$ (or $\Theta_1 = 180°$) and $\phi = 90°$, however, not as low as the ground state energy reference (close-packed, $\phi = 0°$). The favouring of $\Theta_1 = 60$, $120°$ over $\Theta_1 = 0$ at $\phi = 90°$ is due to the twisting effect, and in this case, even a hexagonal lattice (AAA) is favoured over the closed-packed (ABC).

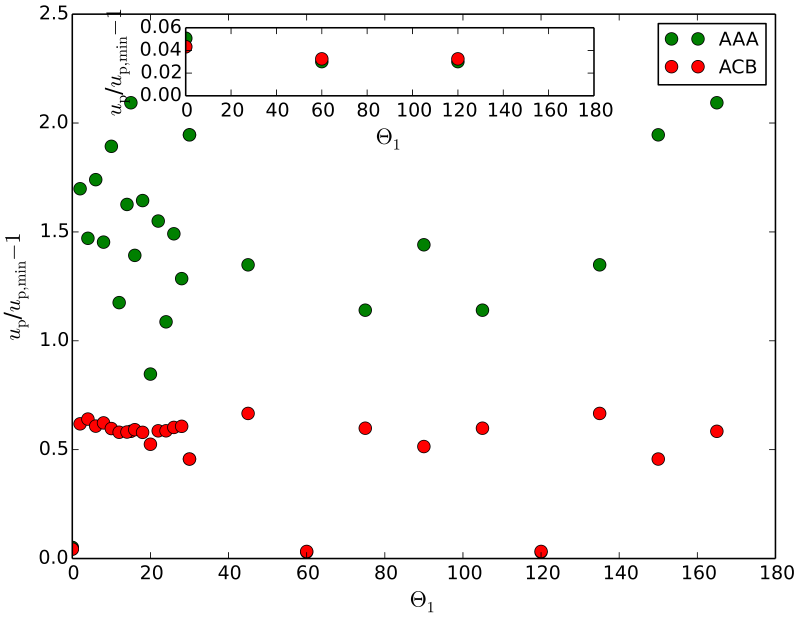

Figure S. 3: Energy per particle difference at various twist angles, Θ_1, at $\phi = 90°$, for lattices AAA and ABC, relative to the ground state (ABC, $\phi = 0°$). The initial angle, Θ_2 $=0$. $\rho^* = 10$ and $\kappa^* = 10$.

Additional energy-minimised crystals

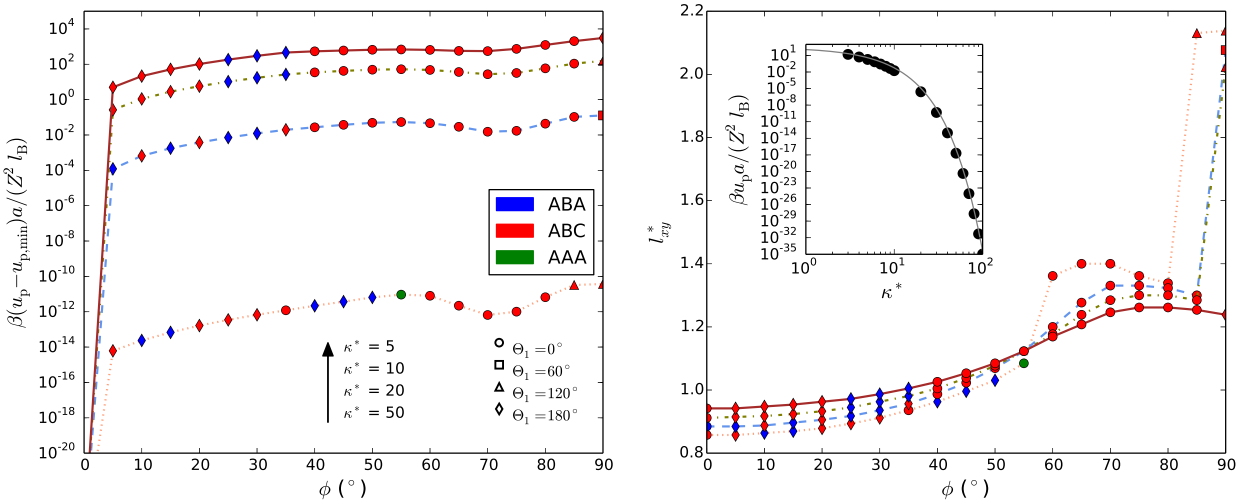

Figure S. 4: Left: Energies per particle for the optimal crystal structures at various tilt angles, ϕ. Right: the reduced lattice dimensions, l^*_{xy}, corresponding to the energies to the left. Inset: energy dependency on the reduced inverse screening length, κ^*, shown for the ground state structure (close-packed, z-axis oriented). The reduced rod density is $\rho^* = 1$. Inset: Energy as a function of κ^*. Line shows the expression $u_p \sim e^{-\kappa^* l^{*c}_{xy}}$, where l^{*c}_{xy} is characteristic (here 0.85) for this ρ^*.

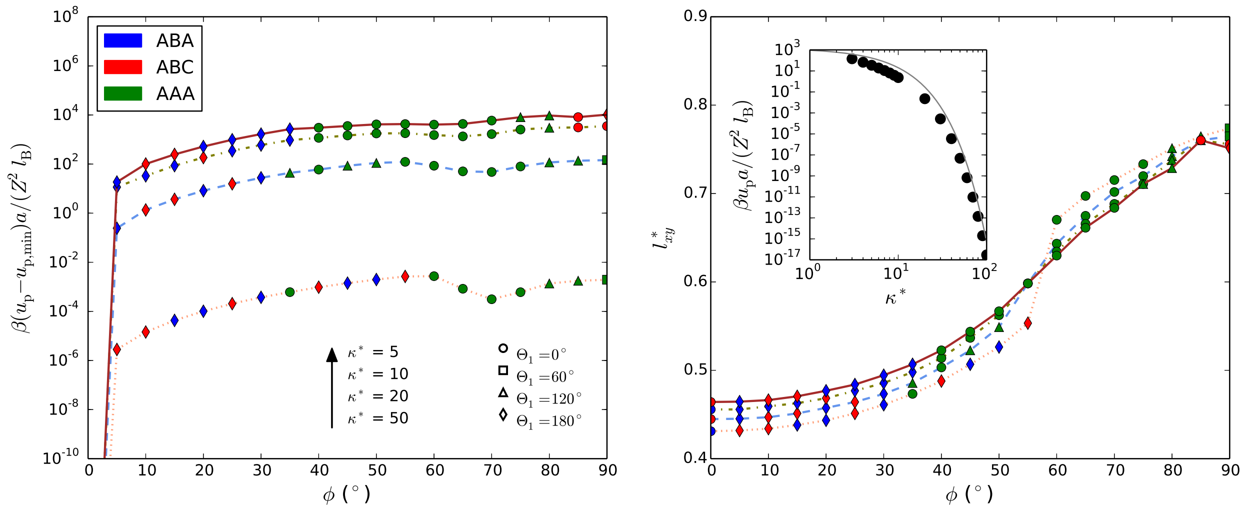

Figure S. 5: Left: Energies per particle for the optimal crystal structures at various tilt angles, ϕ. Right: the reduced lattice dimensions, l^*_{xy}, corresponding to the energies to the left. Inset: energy dependency on the reduced inverse screening length, κ^*, shown for the ground state structure (close-packed, z-axis oriented). The reduced rod density is $\rho^* = 5$. Inset: Energy as a function of κ^*. Line shows the expression $u_\mathrm{p} \sim e^{-\kappa^* l^{*c}_{xy}}$, where l^{*c}_{xy} is characteristic (here 0.43) for this ρ^*.

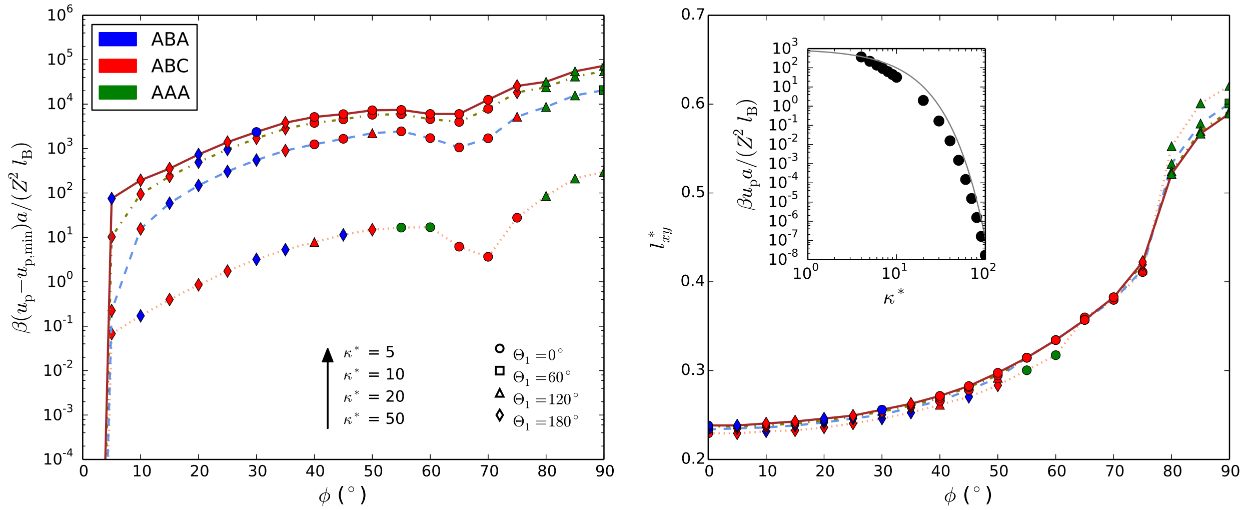

Figure S. 6: Left: Energies per particle for the optimal crystal structures at various tilt angles, ϕ. Right: the reduced lattice dimensions, l^*_{xy}, corresponding to the energies to the left. Inset: energy dependency on the reduced inverse screening length, κ^*, shown for the ground state structure (close-packed, z-axis oriented). The reduced rod density is $\rho^* = 20$. Inset: Energy as a function of κ^*. Line shows the expression $u_\mathrm{p} \sim e^{-\kappa^* l^{*c}_{xy}}$, where l^{*c}_{xy} is characteristic (here 0.23) for this ρ^*.

Monte Carlo simulations, $T = 0$

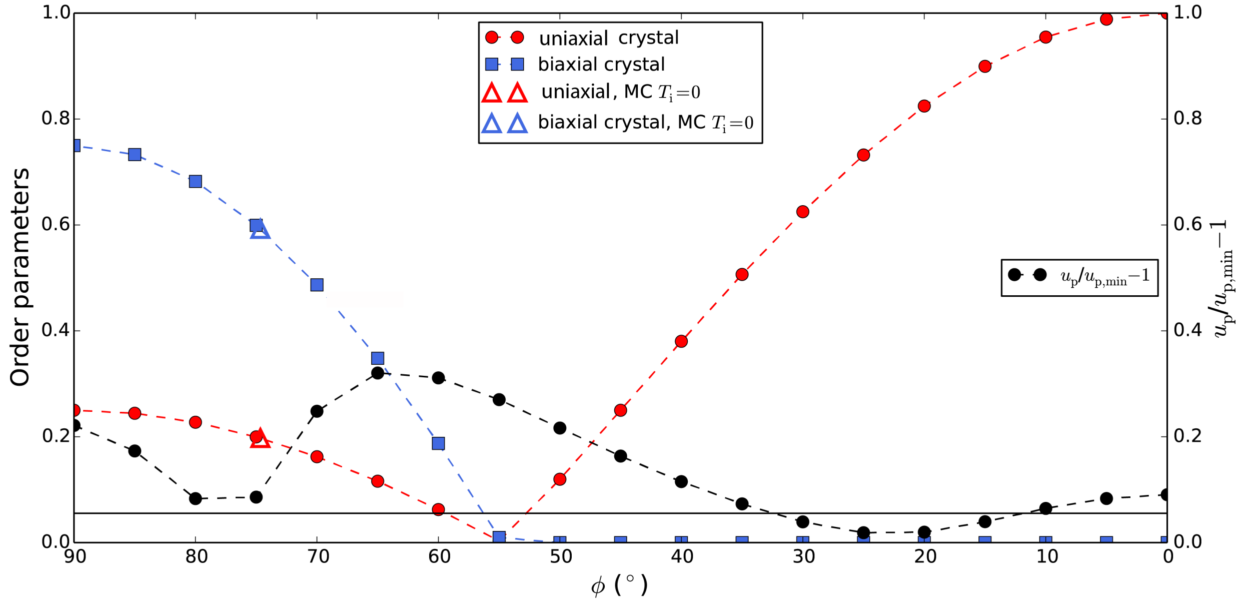

Figure S. 7: Uniaxial and biaxial order parameters, and energy per particle difference relative to the ground state at various ϕ for a AAA-lattice with $\Theta_1 = 120°$. The horizontal line represent the corresponding energy at the end of the temperature sequence of $T_i = 0$ in Figure 6.

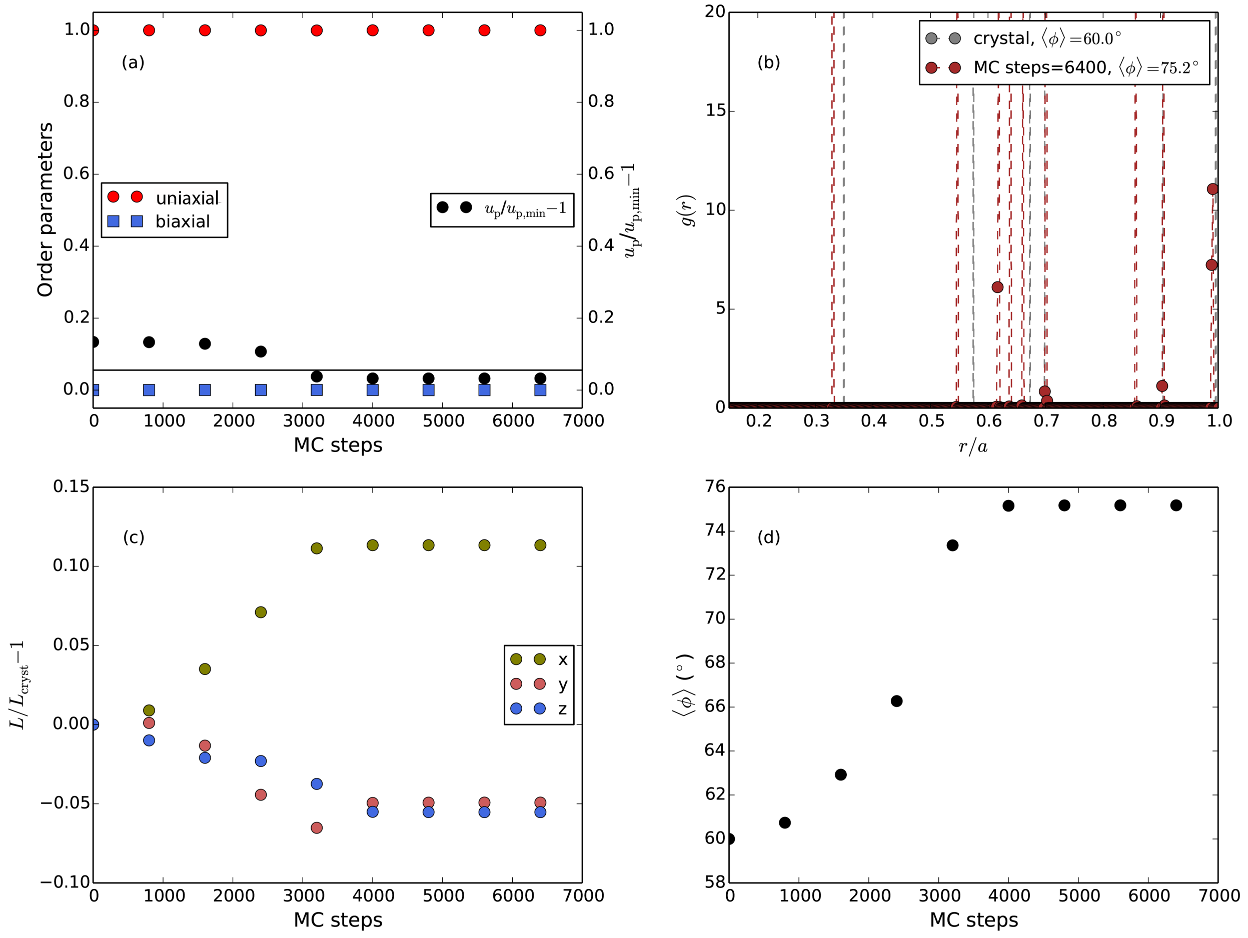

Figure S. 8: Zero-temperature Monte Carlo simulation starting in the crystalline configuration, lattice=AAA, $\Theta_1 = 0°$, $\phi = 60°$. (a): Uniaxial and biaxial order parameters, and energy per particle difference relative to the ground state. The horizontal line represents the corresponding energy from a simulation starting from the heliconical configuration AAA, $\Theta_1 = 120°$, $\phi = 75°$. (b) pair-correlation function. (c): relative change in simulation box dimensions (L_x, L_y, L_z) during the course of the simulation, where $L_{\rm crystal}$ denotes the respective box length (x, y or z) of the initial crystal. (d): average tilt angle (ϕ).
